# Transcriptional responses of ecologically diverse *Drosophila* species to larval diets differing in relative sugar and protein ratios

**DOI:** 10.1371/journal.pone.0183007

**Published:** 2017-08-23

**Authors:** Nestor O. Nazario-Yepiz, Mariana Ramirez Loustalot-Laclette, Javier Carpinteyro-Ponce, Cei Abreu-Goodger, Therese Ann Markow

**Affiliations:** 1 Laboratorio Nacional de la Genomica de Biodiversidad, Irapuato, Guanajuato, Mexico; 2 Department of Biology, University of Maryland, College Park, Maryland, United States of America; 3 Department of Cell and Molecular Biology, University of California San Diego, La Jolla, California, United States of America; CINVESTAV-IPN, MEXICO

## Abstract

We utilized three ecologically diverse *Drosophila* species to explore the influence of ecological adaptation on transcriptomic responses to isocaloric diets differing in their relative proportions of protein to sugar. *Drosophila melanogaster*, a cosmopolitan species that breeds in decaying fruit, exemplifies individuals long exposed to a Western diet higher in sugar, while the natural diet of the cactophilic *D*. *mojavensis*, is much lower in carbohydrates. *Drosophila arizonae*, the sister species of D. mojavensis, is largely cactophilic, but also utilizes rotting fruits that are higher in sugars than cacti. We exposed third instar larvae for 24 hours to diets either (1) high in protein relative to sugar, (2) diets with equal amounts of protein and sugar, and (3) diets low in protein but high in sugar. As we predicted, based upon earlier interspecific studies of development and metabolism, the most extreme differences in gene expression under different dietary conditions were found in *D*. *mojavensis* followed by *D*. *arizonae*. No differential expression among diets was observed for *D*. *melanogaster*, a species that survives well under all three conditions, with little impact on its metabolism. We suggest that these three species together provide a model to examine individual and population differences in vulnerability to lifestyle-associated health problems such as metabolic syndrome and diabetes.

## Introduction

Over the past decades there has been an enormous shift worldwide in the way people eat [1]. One of the most notable components of the dietary shift has been the increase in caloric sweeteners in beverages and packaged foods that already were high in carbohydrates relative to protein [1]. Historically, human populations were accustomed to inconsistent food sources thus resulting in the polygenic “Thrifty Genotype” as proposed by Neel [2] adapted to store calories, when they were abundant, in order to survive lean times. In the transition to consistently high calorie diets, rich in sugars, the Thrifty Genotype responds by producing obesity and metabolic disorders.

Despite this, not everyone who consumes excessive sugars becomes obese or ill. There is considerable genetic variability in how individuals respond to dietary intakes [3]. Detecting genetic variants in metabolic pathways that underlie some sort of resistance to becoming obese or ill, in the presence of an excess of empty calories, may offer insights to novel prevention or treatment approaches. Genome wide association studies already have revealed multiple candidate loci with variant alleles, confirming Neel’s Thrifty Genotype hypothesis [4]. Verification of the function of these genes, however, requires a more empirical approach. Experimental or manipulative studies, unfortunately, are difficult with humans or even with vertebrate model systems owing to factors such as generation time or expense and redundant genes. It is not surprising, therefore, that models such as *Drosophila*, that has many orthologs to human disease genes [5], are gaining popularity in metabolic disease research [6].

Ecologically diverse *Drosophila* species whose genomes have been sequenced [7] show profoundly different responses to identical laboratory larval diets [8]. The most striking differences are reflected in the adult metabolic pools of protein, triglycerides and glycogen, revealing that the species’ genotypes are adapted to natural resources that differ in their relative macro and micronutrients. Species such as the desert endemic *D*. *mojavensis* breeds exclusively in necrotic cactus, a resource low in carbohydrates, while *D*. *melanogaster* is adapted to decaying domestic fruits, typically much higher in their sugar content. These differences make the two species similar to the “Thrifty Genotype” human populations that remained isolated until recently from processed foods (*D*. *mojavensis*) and to a “Western diet” (*D*. *melanogaster*). Indeed, when the two species are grown in isocaloric media that differ in their relative amounts of protein to sugar *D*. *mojavensis* suffers a reduced fitness as well as significant increases in metabolic pools of triglycerides and glycogen, as the relative dietary sugar content increased [9, 10]. *Drosophila mojavensis* has a close relative, *D*. *arizonae*, which although mainly cactophilic, also utilizes decaying fruits such as citrus, making it more of a dietary intermediate between the other two species. Furthermore, these two species can be crossed in the laboratory, allowing for genetic experiments not possible between either species with the more distant relative *D*. *melanogaster*. Previously we tested the prediction that while *D*. *arizonae* would not perform as well as *D*. *melanogaster* on a higher sugar diet, it would be less affected than *D*. *mojavensis* [10]. We reasoned that if *D*. *arizonae* is indeed more accustomed to consuming sugar in nature, it should have higher larval to adult survival as well as lower perturbation of metabolic pools than its sister species *D*. *mojavensis* on the diets with higher sugar contents. While *D*. *arizonae* in fact exhibits a reduced survival on a low-protein/high sugar diet, sufficient numbers do survive to adulthood to reveal that their metabolic pools of glycogen and triglycerides also are elevated as sugar increases relative to protein [10].

The metabolic pool data reported for adults of these species [9, 10] represent the cumulative effect of growth and survival in different environments rather than more immediate metabolic or transcriptomic responses. Therefore, we wished to determine what short-term changes in gene expression, if any, are observed when each of the three species is confronted with increasing amounts of sugar relative to protein. We utilized early third instar larvae because they are more resistant to any damage from transferring them to various diets and because they are larger and yield more material for analyses. We thus exposed third instar larvae to three experimental diets for 24 hours and then examined their transcriptomic responses to the diets. We predicted that if changes are observed, they would occur in the following order of severity of perturbations in gene expression as the diets increase in relative sugar levels: *D*. *mojavensis* > *D*. *arizonae > D*. *melanogaster*.

## Methods

### Flies

We utilized two isofemale strains of each species: *D*. *melanogaster* from San Diego, USA (Iso-SD1 and Iso-SD3), one *D*. *arizonae* strain from Sinaloa, México (Iso-13-3) and one from Queretaro, Mexico (Iso-15-7) and two strains of *D*. *mojavensis* from La Paz, B.C.S., México (Baja-1C and Baja-1F). All stocks, prior to use in experiments, were grown at room temperature (~25°C) in standard banana-opuntia media on a 14:10 L:D cycle. To obtain larvae for development or transcriptomic studies, sexually mature adults were placed in yeasted egg collecting chambers (Genesee Scientific) and the embryos were allowed to develop to early third instar at which time they were transferred to the experimental diets for 24 hours. Experiments were initiated and larvae harvested for RNA extraction at the same time mid-day in all replications in order to avoid any effects of circadian cycles in gene expression.

### Experimental diets

Isocaloric artificial diets were prepared [9]: a diet with a high ratio of protein:sugar or HPLS, one with equal protein:sugar or EPS, and a diet with low protein:sugar or LPHS (S1 Table). Protein was supplied by active dry yeast and sugar by sucrose, complemented with yellow cornmeal and agar. All ingredients were mixed and boiled, with methylparben added as a fungal inhibitor.

### Transcriptomic response to experimental diets

Early third instar larvae from two different isofemale strains/species were transferred to the experimental diets for 24hrs (n = 30/vial). Larvae then were removed from their experimental foods, washed twice with distilled water and placed into 1.5μl tubes to be rinsed twice with 1X PBS. All liquid was then removed and larvae were frozen (-70°C) until RNA extraction. Frozen samples were homogenized with Tri-Reagent using Teflon homogenizers and extracted using Direct-zol^TM^ RNA MiniPrep extraction kit (Zymo Research) according to the manufacturer’s protocol. Three aliquots of each sample were taken, one to measure RNA concentration in NanoDrop (Thermo Scientific), another for analyses in a 1% agarose gel, and one for the sequencing core facility at LANGEBIO. Libraries were prepared with TruSeq® RNA Sample Preparation Kit v2 (Illumina), selecting only polyA mRNAs and synthetizing double stranded cDNAs to attach to the Illumina adapters. Library size and quality were measure by Bioanalyzer (Agilent Technologies) and sequenced in a 2 X 100 pair-end read format on a HiSeq^TM^ 2000 Sequencing System (Illumina).

### Transcriptome analyses

Transcriptome analysis was performed using the TopHat-Cufflinks pipeline [11]. Correlations between replicates were high, 97–98% and are presented in S3 Table. Reference genomes were indexed with “bowtie2-build”, using genome versions: r6.04 for *D*. *melanogaster* (FlyBase), first release for *D*. *arizonae* [12] and r1.3 for *D*. *mojavensis* (FlyBase). Paired-end reads were mapped with “bowtie2/tophat2” (S2 Table) and showed a good correlation between replicates (S3 Table). For *D*. *arizonae*, “cufflinks” and “cuffmerge” were applied to generate a GTF file, containing the locations of predicted transcripts.

### Statistical analyses and graphs

Differential expression was calculated with “cuffdiff2”, using the BAM files that TopHat generated, with default settings and FDR ≤ 0.05 [11]. Graphs were generated by the cummerbund R package [13].

### Gene function annotation for differentially expressed genes

Sequences of differentially expressed transcripts in *D*. *arizonae* were blasted with the KEGG Orthology-Based Annotation System (KOBAS 2.0 web tool in http://kobas.cbi.pku.edu.cn) with the “Annotate” program, using nucleotide sequences as input, with default settings (E-value ≤ 1e-08) and *D*. *mojavensis* as a reference. NCBI_Gene_IDs of *D*. *melanogaster*’s orthologs for *D*. *arizonae* and *D*. *mojavensis* (S4 Table) were used in the web tool of Gene Ontology Consortium (http://geneontology.org) comparing with the complete set of data for biological process (BP), molecular function (MF) and cellular component (CC). Enrichment of KEGG pathways was made with KEGG_IDs or NCBI_GIs of *D*. *mojavensis* (S4 Table) with the “Identify” program using as input the exit data file of “Annotate”. All data have been placed in the GEO repository can be accessed through series record GSE101664, through https://www.ncbi.nlm.nih.gov/geo/info/linking.html.

### Validation of differentially expressed genes by RT-qPCR

Using RT-qPCR we validated 10 genes from *D*. *arizonae* and 15 genes from *D*. *mojavensis*. Nine of these were shared genes in both species, significantly differentially expressed according to our RNA-Seq results, when the two extreme diets were compared. Five more were differently expressed only in *D*. *mojavensis*. We designed the primers aligning the sequences of each gene of both species with Geneious (Biomatters), to generate a PCR fragment with recommended length of 100-200bp (S5 Table). We used 18S gene as internal control [14]. Standard protocols of retrotranscription and qPCR were followed, generating single strand cDNAs with SuperScript® lll Reverse Transcriptase (Invitrogen), adding a mix of all reverse primers of target genes to RNA samples and incubating at 55°C during 3 hr. Primer efficiencies are given in S5 Table. Four technical replicates of qPCR for each sample were performed in a 7500 Real Time System (Applied Biosystems). We established the optimal conditions of each set of primers to ensure that the end-point PCR generated only a single band product and dissociation curves showed a single amplicon.

## Results

### Transcriptome

**Fig 1:**
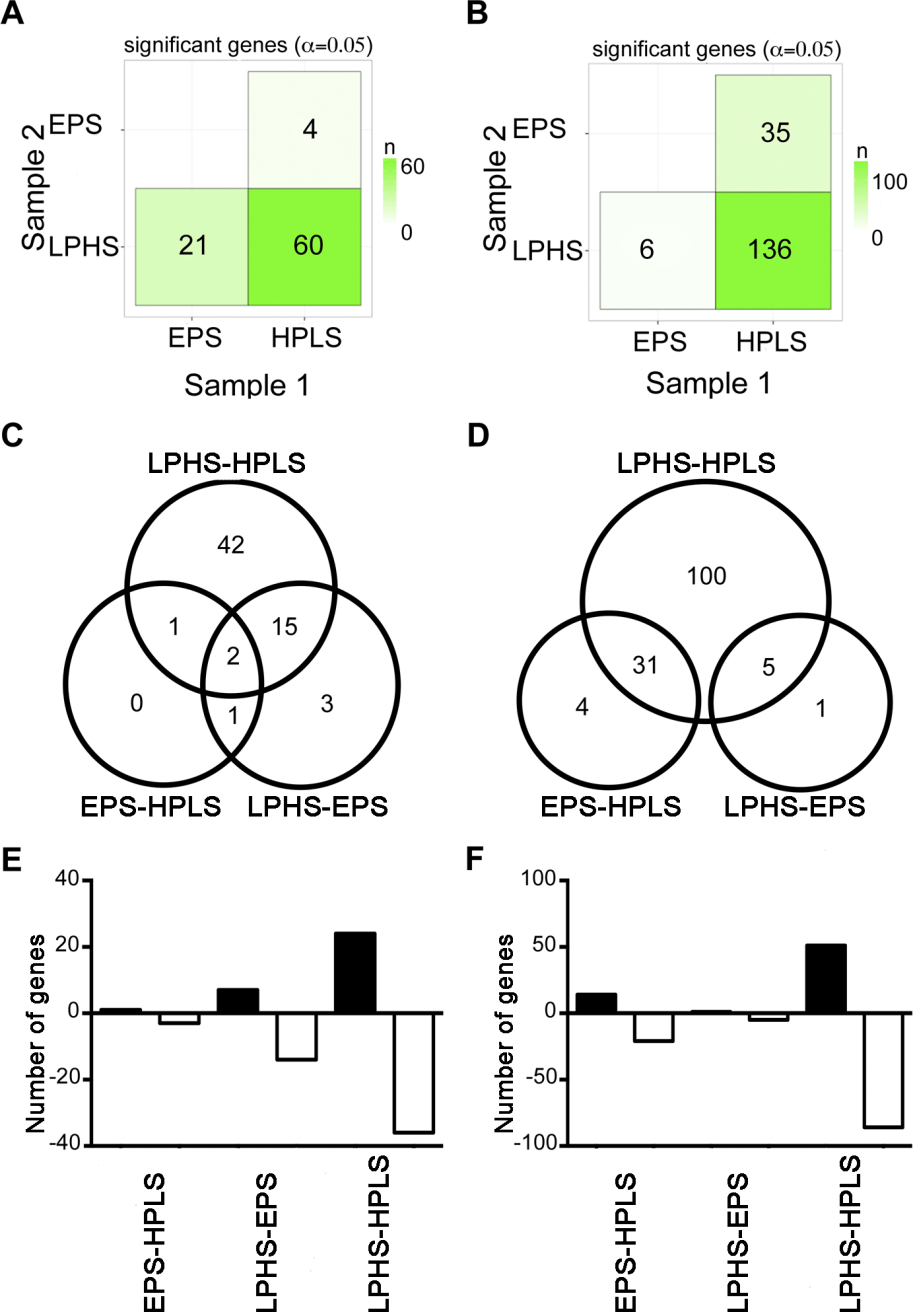
Differentially expressed genes between diets in *D*. *arizonae* and *D*. *mojavensis*. Third instar larvae were exposed to different diets during 24hrs and RNA-Seq analysis was performed with TopHat-Cufflinks (FDR<0.05). (A, C and E) Analysis of differentially expressed genes in *D*. *arizonae*. (B, D and F) Analysis of differentially expressed genes in *D*. *mojavensis*. (A-B) Matrix analysis showing the number of genes that changed between diets. (C-D) Venn diagrams showing the number of genes that were significantly different in more than one paired comparisons. (E-F) Number of genes that were up-regulated (black bars) and down-regulated (white bars) in each paired comparison between diets.

Pairwise comparisons among the three diets for larval *D*. *melanogaster* yielded no significant differences in gene expression. The number of differentially expressed genes among the diets for *D*. *arizonae* and *D*. *mojavensis* are presented in Fig 1. *Drosophila mojavensis* showed the greatest number of differences, and for both species, the greatest number of genes showing differential expression emerged when comparing the low-protein/high-sugar (LPHS) and high-protein/low-sugar (HPLS) diets (Fig 1A and 1B). While some of the genes differentially expressed between the LPHS and HPLS diets overlapped with differentially expressed genes in another comparison (Fig 1C and 1D), the majority were confined to the comparison between the most contrasting diets and were mostly down regulated in LPHS in relation to HPLS (Fig 1E and 1F).

**Fig 2:**
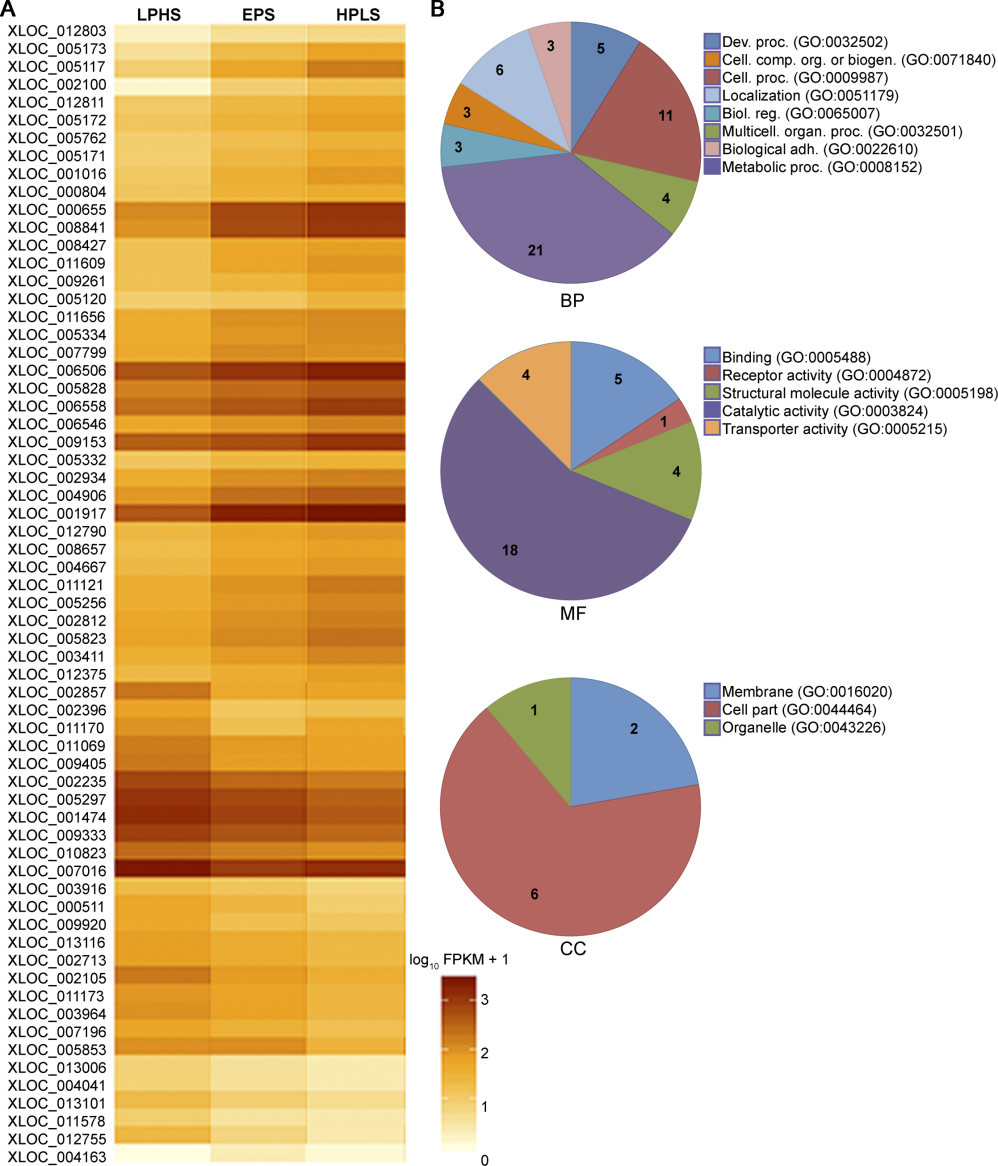
Heat map of all differentially expressed genes of *D*. *arizonae* and their associated GO terms. A) Heat map of the 64 differentially expressed genes analyzed by TopHat-Cufflinks. Color intensity represents the mean of gene expression of the Cufflinks-determined FPKM values for two replicates in each treatment (FDR<0.05). B) Pie charts of functional annotation of affected genes through diets. Analysis was done using the web tool of Gene Ontology Consortium (http://geneontology.org). BP = biological process, MF = molecular function, CC = cellular components. Numbers inside the pie charts are the number of genes associated to each term, and genes that belong to more than one term were counted also in those respective categories.

Sixty-four differentially expressed genes were detected in *D*. *arizonae* when reared on different diets (Fig 2A). When comparing increasing levels of sugar (EPS-HPLS, LPHS-EPS or LPHS-HPLS), twenty-six genes were up-regulated and the remaining 38 were down-regulated (S6 Table). The functional annotation of the differentially expressed genes in *D*. *arizonae* is presented in Fig 2B. We were unable to identify 25 genes in the analysis because 20 have no *D*. *melanogaster* ortholog and five had an ortholog but with no annotation (Table 1). We found information of the remaining 39 genes in the Gene Ontology data base assigned to eight biological process (BP) categories: “cellular process”, “biological adhesion”, “multicellular organismal process”, “developmental process”, “localization”, “biological regulation”, “cellular component organization or biogenesis” and “metabolic process”. The largest component was “metabolic process” with 21 genes. There were 32 genes that had a GO term from the molecular function (MF) ontology, with five terms: “binding”, “receptor activity”, “structural molecule activity”, “transporter activity” and “catalytic activity”. This last term was assigned to 18 of the 32 genes. Only nine genes had a cellular component (CC) term, including: “membrane”, “organelle” and “cell part”.

**Table 1:** Statistics of functional annotation of differentially expressed genes.

	Reference list (D. melanogaster)	Input*
*D*. *arizonae*		
**Non-orthologous:**	-	20
**Unmapped IDs:**	-	5
**Mapped IDs:**	13690	39
***D*. *mojavensis***		
**Non-orthologous:**	-	26
**Unmapped orthologous:**	-	20
**Mapped orthologous:**	13690	95

*Analysis was done with *D*. *melanogaster*’s ortholog IDs using the web tool of Gene Ontology Consortium (http://geneontology.org).

**Fig 3:**
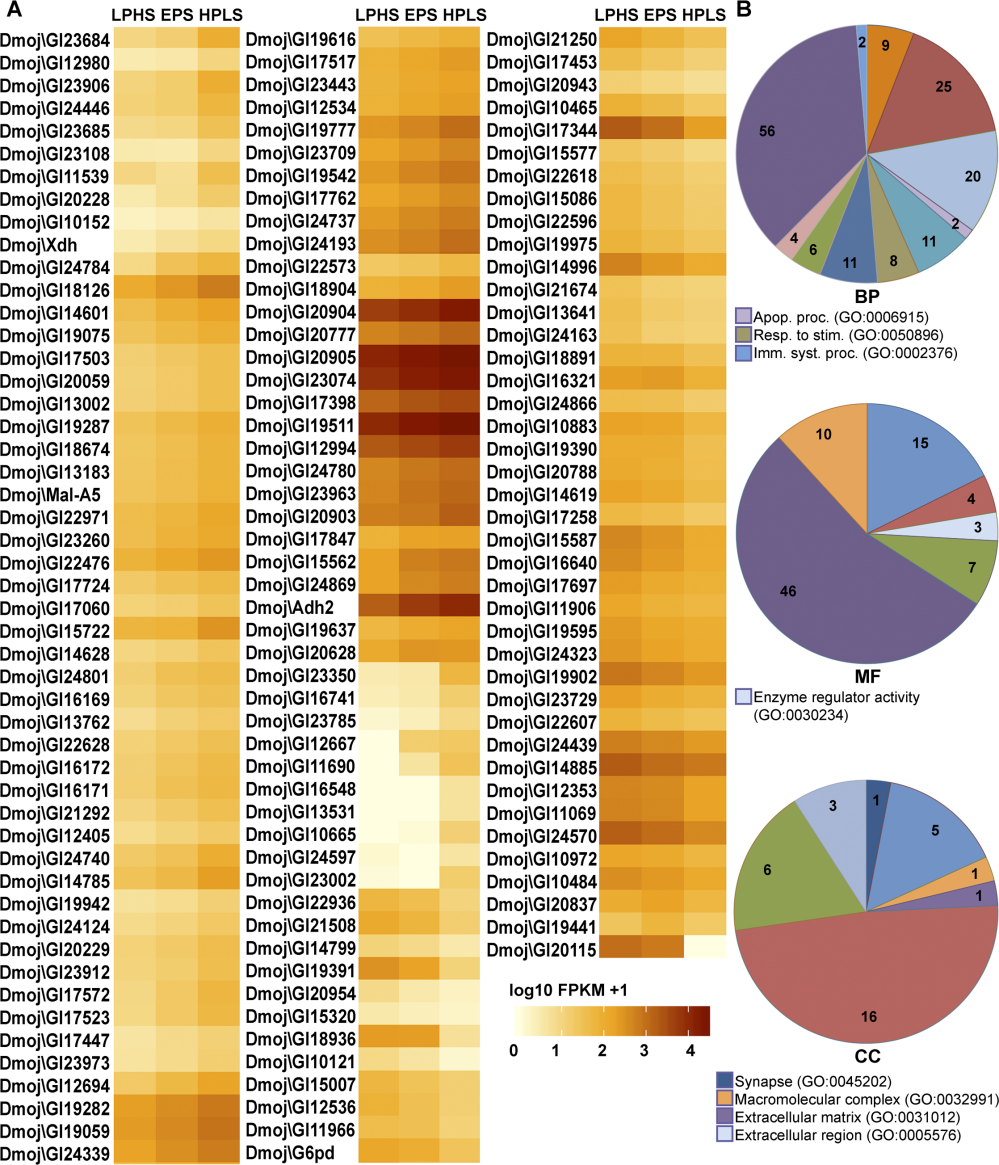
Heat map of all differentially expressed genes of *D*. *mojavensis* and their associated GO terms. A) Heat map of the 141 differentially expressed genes analyzed by TopHat-Cufflinks. Color intensity represents the mean of gene expression of the Cufflinks-determined FPKM values for two replicates in each treatment (FDR<0.05). B) Pie charts of functional annotation of affected genes through diets. Analysis was done using the web tool of Gene Ontology Consortium (http://geneontology.org). BP = biological process, MF = molecular function, CC = cellular components. Color codes are the same as Fig 3 for each chart. Numbers inside the pie charts are the number of genes associated to each term, and genes that belong to more than one term were counted also in those respective categories.

Since *D*. *mojavensis* had the worst performance in the higher sugar diets [10] it was not surprising that we detected 141 differentially expressed genes (Fig 3A). Similarly to *D*. *arizonae*, fewer genes were up-regulated (52) than down-regulated (89) (S7 Table). The functional annotation of the differentially expressed genes in *D*. *mojavensis* is presented in Fig 3B. *Drosophila melanogaster* orthologs and functions were found for 95 genes in the Gene Ontology data base. Twenty had orthologs with no functions and 26 had no orthologs (Table 1). In *D*. *mojavensis*, the BP categories present were: “cellular process”, “biological adhesion”, “multicellular organismal process”, “developmental process”, “localization”, “biological regulation”, “cellular component organization or biogenesis”, “metabolic process”, “apoptotic process”, “response to stimulus” and “immune system process”, but “metabolic process” remained the largest term with 56 genes. “Enzyme regulator activity” was the MF term that appeared in addition to those *D*. *arizonae*, but “catalytic activity” for a second time was the more representative with 46 genes (Fig 3B) despite only 85 genes have this kind of terms. Again, fewer genes were assigned a CC term, so for the 33 genes with this kind of annotation the categories were: “membrane”, “organelle”, “cell part”, “synapse”, “macromolecular complex”, “extracellular matrix” and “extracellular region”.

Expression of 26 genes was affected in both species in higher sugar diets, of which seven were up- and 19 down-regulated (red genes in S6 and S7 Tables).

### Validation of differentially expressed genes

**Fig 4:**
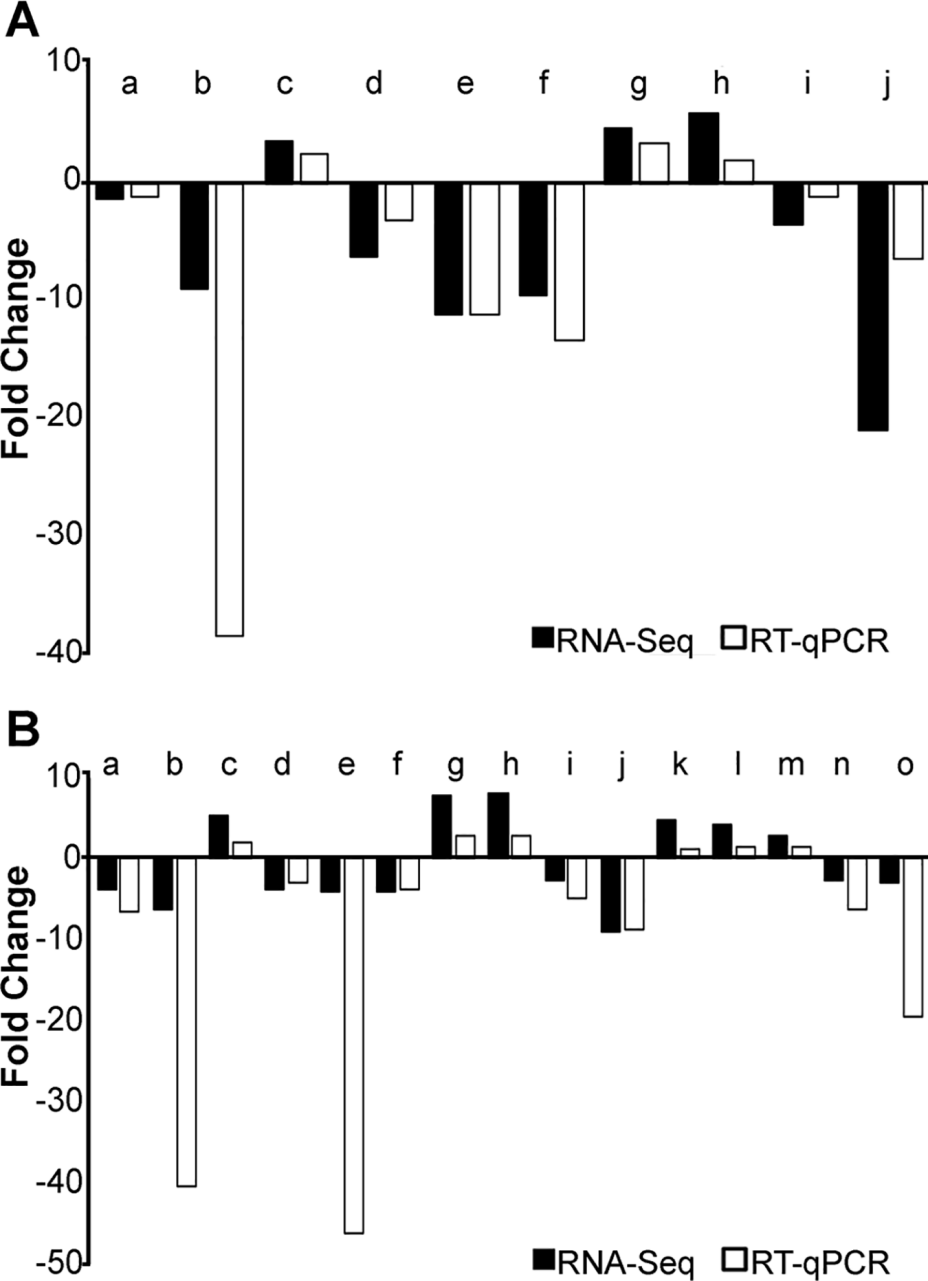
Validation of differentially expressed genes by RT-qPCR. A) Differentially expressed genes between more extreme diets (LPHS-HPLS) of *Drosophila arizonae*, using 18S transcript as internal control. Genes are: a = XLOC_001916 (*Adh1*), b = XLOC_001917 (*Adh2*), c = XLOC_007196 (*G6pd*), d = XLOC_012803, e = XLOC_008841, f = XLOC_000655, g = XLOC_011578, h = XLOC_000511, i = XLOC_008657, j = XLOC_005117. B) Differentially expressed genes between more extreme diets (HPLS-LPHS) of *Drosophila mojavensis*, using 18S transcript as internal control. Genes are: a = *Adh1*, b = *Adh2*, c = *G6pd*, d = *Xdh*, e = GI11539, f = GI15562, g = GI20954, h = GI21508, i = GI23443, j = GI23906, k = GI14996, l = GI15007, m = GI19975, n = GI20777, o = GI23074.

We validated a selection of the differentially expressed genes by RT-qPCRs (Fig 4). We assayed 10 *D*. *arizonae* and 15 *D*. *mojavensis* genes: nine that were shared between extreme diets of both species, *Adh1* and five more solely in *D*. *mojavensis* (Table 2). All genes that were differentially expressed by RNA-Seq in *D*. *arizonae* and *D*. *mojavensis* were confirmed to change in the same direction according to RT-qPCR.

**Table 2:** Genes validated by RT-qPCR. Nine genes were differentially expressed by RNASeq and *Adh1* was used for comparison with *Adh2*.

*Shared genes assayed in both species*		*Genes assayed only in D*. *mojavensis*	
*D*. *arizonae* gene ID	*D*. *mojavensis* gene ID		
XLOC_001916	Adh1	GI14996	
XLOC_001917	Adh2	GI15007	
XLOC_007196	G6pd	GI19975	
XLOC_012803	Xdh	GI20777	
XLOC_008841	GI11539	GI23074	
XLOC_000655	GI15562		
XLOC_011578	GI20954		
XLOC_000511	GI21508		
XLOC_008657	GI23443		
XLOC_005117	GI23906		

In addition, based upon primers designed to differentiate the paralogs *Adh1* and *Adh2* (“a” and “b” in Fig 4A and 4B), we determined that *Adh2* but not *Adh1*, was the differentially expressed paralog. Furthermore, RT-qPCR confirmed that of all the differentially expressed genes assayed, *Adh2* had one of the greatest fold changes in the HPS-LPS comparison in both species. Some transcripts were more sensitive to RT-qPCR, such as *Adh2* in both species (“b” in Fig 4A and 4B), or GI11539 and GI23074 in *D*. *mojavensis* (“e” and “o” in Fig 4B). On the other hand, XLOC_005117 (“j” in Fig 4A) in *D*. *arizonae* was less sensitive. Sensitivity can reflect the internal control used for RT-qPCR (18S gene), but despite these subtle qPCR sensitivity differences, all differentially expressed genes in the RNA-Seq were confirmed by RT-qPCR.

## Discussion

As we predicted based upon the effects of the diets on the long-term development and metabolism of the three species [10] the greatest changes in gene expression were observed in *D*. *mojavensis* followed by *D*. *arizonae*. While both species are adapted to low carbohydrate cactus resources, *D*. *arizonae* is also associated with rotting domestic fruits, like *D*. *melanogaster*, and thus is better adapted than *D*. *mojavensis* to deal with excess sugar in their diet. It was no surprise, therefore, that fewer expression changes appeared in *D*. *arizonae* compared to *D*. *mojavensis* when dietary sugar was increased. Thus *D*. *arizonae* can be considered somewhat intermediate between the Thrifty Genotype of *D*. *mojavensis*, and the genotype of *D*. *melanogaster*, which as a fruit breeder, is better adapted to a “Western Diet”.

For *D*. *melanogaster*, 24 hours of feeding by third instar larvae produced no significant changes in gene expression. This was somewhat surprising given that increased metabolic pools of triglycerides and glycogen were observed in adult flies having been reared as larvae on higher sugar diets [9]. On the other hand, the 24 hours exposure here could either have been insufficient to significantly perturb gene expression or the developmental stage tested was not the most sensitive to these diets. Many genes that were affected in high sugar diets and detected by microarray analysis [15,16] were not affected in our study in *D*. *melanogaster*, likely because the proportion of sugar in these earlier studies was higher and in more accessible forms. Zinke *et al*. [15] exposed larvae to filter paper saturated with a 20% sucrose solution and Musselman *et al*. [16] used a 1M sugar concentration.

On the other hand, the two cactus breeders *D*. *arizonae* and *D*. *mojavensis*, less accustomed to sugar than the fruit-breeding *D*. *melanogaster*, showed large perturbations in expression, especially between the most extreme two diets (60 and 136 genes respectively). The majority of differentially expressed genes in *D*. *arizonae* and *D*. *mojavensis* belonged to the BP-GO term “metabolic process” and the MF-GO term “catalytic activity” and we therefore focus our discussion on this set of genes. First we will discuss those changes common to both species as these likely reflect more conserved metabolic responses in general. While we focus our discussion on particular sets of genes, the particular metabolic pathways (KEGG) in which these differentially expressed genes participate can be visualized suing the hyperlink associated with each pathway listed in our supplementary materials (S8 Table)

### Genes that changed expression in *D*. *arizonae* and *D*. *mojavensis*

#### Carbohydrate metabolism

Three of the 26 genes upregulated in both species in higher sugar diets are related to carbohydrate metabolism (Table 3). Two of these overexpressed genes, an UDP-glucuronosyltransferase or UGT (XLOC_011578 and GI20954) and Trehalose 6-phosphate phosphatase or T6PP (XLOC_002105 and GI23729) belong to the “Starch and sucrose metabolism” (dmo00500, KEGG pathway). CG17323, the *D*. *melanogaster* ortholog of this UGT is expressed in the fat body and was overexpressed in high sugar diets in *D*. *melanogaster* [15]. T6PP is required to form trehalose by Trehalose 6-phosphate [17]. Trehalose, the principal disaccharide present in hemolymph, is synthesized before glycogen in many insects [18]. Elevated levels of trehalose occur in flies fed on high sugar diets [16]. The other gene upregulated in high sugar encodes G6pd (XLOC_007196 and Dmoj\G6pd, a key enzyme in the “pentose phosphate pathway” (dmo00030, KEGG pathway) (S8 Table), required to generate NADPH for lipid biosynthesis [19]. G6pd also was up-regulated in *D*. *melanogaster* fed high sugar diets in Zinke *et al*. [15]. This upregulation of these three genes seems to be a highly conserved response to high sugar in *Drosophila*.

**Table 3:** Expression changes of shared genes that were differentially expressed in *D*. *arizonae* and *D*. *mojavensis* in LPHS-HPLS.

*D*. *arizonae* (Gene ID)	*D*. *mojavensis* (Gene symbol)	Enzyme/protein*	*D*. *arizonae* Log_2_FC	*D*. *mojavensis* Log_2_FC
Carbohydrate metabolism				
*XLOC_002105*	*Dmoj\GI23729*	T6PP	2.3	1.6
*XLOC_011578*	*Dmoj\GI20954*	UGT	2.2	2.8
*XLOC_007196*	*Dmoj\G6pd*	G6PD	1.8	2.4
Lipid metabolism				
*XLOC_000511*	*Dmoj\GI21508*	ETNPPL	2.6	3.0
*XLOC_005332*	*Dmoj\GI24124*	Carboxilesterase	-1.5	-1.7
*XLOC_001917*	*Dmoj\Adh2*	ADH	-3.2	-2.6
Development and growth				
*XLOC_011069*	*Dmoj\GI15007*	d4E-BP	1.4	2.0
*XLOC_012803*	*Dmoj\Xdh*	XDH	-2.6	-1.9
*XLOC_008841*	*Dmoj\GI11539*	Fbp1	-3.5	-2.0
Amino acid synthesis and utilization				
*XLOC_008657*	*Dmoj\GI23443*	AMT	-1.8	-1.4
*XLOC_002100*	*Dmoj\GI23785*	SARDH	-5.1	-4.0
Retinol metabolism				
*XLOC_011578*	*Dmoj\GI20954*	UGT	2.2	2.8
*XLOC_001917*	*Dmoj\Adh2*	ADH	-3.2	-2.6

*T6PP = trehalose 6-phosphate phosphatase, UGT = UDP-glucuronosyltransferase, G6PD = Glucose 6-phosphate dehydrogenase, ETNPPL = Ethanolamine-phosphate phospholyase, ADH = Alcohol dehydrogenase, d4E-BP = Eukaryotic translation initiation factor 4E binding protein, XDH = Xantine dehydrogenase, AMT = Aminomethyltransferase, SARDH = Sarcosine dehydrogenase.

#### Lipid metabolism

Another three genes common to both species are involved in lipid metabolism (Table 3). ADH is known to control the flux from ethanol to lipids, specifically triglycerides in *Drosophila* larvae [20], and is especially sensitive to sugar concentrations [21]. In *D*. *mojavensis* and *D*. *arizonae* the ADH gene is duplicated, with *Adh1* and *Adh2* being differentially expressed during development. Expression of *Adh1* begins in the embryo and early larval stages, but disappears in adults except in the ovaries. *Adh2* appears during late third instar larvae and continues in the adult [22]. While diet had no effect on the expression of *Adh1*, at the time *Adh2* begins to be expressed, it was down-regulated by high sugar diet (XLOC_001917 and GI17643). *Adh2* thus is likely involved in different functions from *Adh1*, since their expression profiles were not affected in the same way. An Ethanolamine phosphate phospholyase (XLOC_000511 and GI2150), known to be involved in *Drosophila* lipid droplet storage [23] was up-regulated, while an ortholog of *α-Est2* (XLOC_005332 and GI24124), also involved in lipid droplet storage in fat body [24] as down-regulated as well.

#### Development and growth genes

Two genes down-regulated in both species in response to the LPHS diet are connected to the action of juvenile hormone (JH) and ecdysone. Down-regulation of XDH (XLOC_012803 and Dmoj\Xdh) was also reported in *D*. *melanogaster* under high sugar conditions [15]. XDH, encoded by *rosy* in *D*. *melanogaster*, functions in purine metabolism, converting xanthine to uric acid. Interestingly, *rosy* mutants are insensitive to JH, suggesting a connection between this gene and delayed growth and development [25]. The *D*. *melanogaster* ortholog, *fbp1*, of the second down-regulated gene, GI11539 and XLOC_008841 is preferentially expressed in fat body [26] and is a direct target of the ecdysone receptor, possibly mediating ecdysone effects [27].

Up-regulated in both species in response to the LPHS diet were the orthologs of *Thor*: the genes that encode d4E-BPs in *D*. *mojavensis* (GI14996 and GI15007) and *D*. *arizonae* (XLOC_011069). *Thor* acts in the insulin pathway, sensing nutrient availability and regulating growth by inhibiting translation [28] and also was up regulated in *D*. *melanogaster* exposed to high sugar [15].

An intriguing connection thus appears to exist between development and growth that involves JH, ecdysone and the insulin pathway. JH impairment is suggested to affect the repression of ecdysone, slowing growth though changes in the insulin pathway [29]. ADH and UGT (down- and up-regulated respectively) while related to retinol metabolism (dmo00830, KEGG pathway) (S8 Table) are also related to ecdysone secretion, mediating retardation of pupation in larva of *Drosophila* that irradiated with X-rays [30]. Disruption of this JH-Ecdysone relationship in the low protein-high sugar diet could explain why *D*. *mojavensis* larvae remained in the LPHS food for a long period but failed to pupate [10]. Delayed development also has been linked to dietary changes in another cactophilic species, *D*. *buzzatii* [31], although it remains to be explored if there are any underpinnings common to our study.

#### Genes that change only in D. mojavensis

Even following only 24 hours of exposure to the LPHS diet, large number of expression changes occurred in *D*. *mojavensis*. Given the natural history of this species and its poor performance on the LPHS diet [10], a larger effect at the transcriptional level is not surprising, especially for genes involving processes such as apoptosis. A large number of expression changes clearly are related to metabolic stress in key pathways. Carbohydrate metabolism genes, for example, appear particularly sensitive to the LPHS diet. For example, three additional UGTs (GI17523, GI20943, and GI22628) in the “starch and sucrose metabolism” category (dmo00500, KEGG pathway) (S8 Table) were down-regulated (S7 Table), as were alpha-glucosidase (Mal-A5 or GI18697) [32] and an Hexokinase (GI19942). *Hex-C* (ortholog of GI19942) is a key regulator of glycolysis, and was also down-regulated in higher sugar diets in *D*. *melanogaster* [16], but there are other Hexokinases that can supply its function [33,16]. Furthermore, 6-phosphogluconate dehydrogenase (GI11069), another enzyme of the pentose phosphate pathway was up-regulated.

Differential regulation of lipid metabolic genes was also observed in *D*. *mojavensis*. These changes involved a 3,2-trans-enoyl-CoA isomerase (GI17724), an acyl-CoA dehydrogenase or ACAD (GI19075), two stearoyl-CoA desaturases or SCDs (GI10484 and GI24323), a wax ester synthase/diacylglycerol o-acyltransferase (GI17060) and a fatty acid hydroxylase (GI14601). Similar mis-regulation of an ACAD and one SCD (CG9743, the ortholog of GI24323) was also reported in flies with insulin resistance in *Drosophila melanogaster* [16]. Importantly, GI19975, the ortholog of *sug* was over-expressed in the higher sugar diets used here as well as in the Zinke et al [15] in *D*. *melanogaster*. *sug* encodes a zinc finger transcription factor that negatively regulates a set of enzymes involved in fat catabolism [15]. Gamma-butyrobetaine dioxygenase (GI19441), which is required for synthesis of carnitine [34] was also up-regulated. Carnitine has been reported to neutralize the deleterious effect of excess of glucose, in addition to its basic function of promoting beta-oxidation [35, 36] and is associated with prevention of obesity [37]. Also, modification of lipid mobilization is implied because *mtp*, the ortholog of GI17847, is required for the formation of apoB-family lipoproteins (Lpp and LTC) in *Drosophila* [38,39].

The observed changes in expression of lipid metabolism genes in *D*. *mojavensis* suggest regulating the metabolism of fats in two ways. On one hand, catabolism of fats can be slowed by down-regulation of GI17724 and GI19075, and up-regulation of the *sug* ortholog. At the same time, however, their synthesis may be promoted by up-regulation of GI10484 and GI24323.

In summary, many genes that changed expression in the LPHS diet in *D*. *arizonae* and *D*. *mojavensis*, belong to the same functional categories. A number of these genes were also affected in *D*. *melanogaster* in previous studies in which flies were exposed to high sugar [15, 16]. Thus many genes involved in metabolic response to high sugar diets are common to all three species, with those species less adapted to dietary sugar, *D*. *arizonae* and especially *D*. *mojavensis*, being more sensitive. The growth delays in *D*. *arizonae* and *D*. *mojavensis* [10] likely are mediated by the insulin pathway mis-regulation of JH and ecdysone. Observed metabolic changes in *D*. *mojavensis* (down-regulation of glycolysis and up-regulation of pentose phosphate pathway, fatty acid and trehalose synthesis) are related to the development of insulin resistance [16]. Although the number of genes affected in each category differ somewhat between *D*. *arizonae* and *D*. *mojavensis*, a core set of alterations potentially control flux in the relevant biochemical pathways [40].

Our experiments, taken with previous results, suggest the existence of core genes to the different diets, but with different sensitivities to dietary perturbations depending upon the conditions of exposure. The situation is not unlike what we might expect from humans of different genetic backgrounds. Future steps could examine the regulatory aspects of these core genes, either by differences in their regulatory sequences or by noncoding RNAs. The genes could also vary in their coding sequence in ways that influence their metabolic efficiencies. Metabolic responses to diets in humans are clearly complex and thus likely controlled by multiple genes and multiple mechanisms regulating the function of those genes. These flies thus appear to present appealing models to examine the roles of various response mechanisms to diets differing in quality.

The value of the conservation of metabolic functions and molecular pathways between flies and humans has been recognized for some time [41]. Here we show that the evolutionary differences among *Drosophila* species appear to mimic the differences among human populations adapted to Western or indigenous or ancient diets based on the Thrifty Genotype. When there is a mismatch between the diet and the genotype, the genes that change expression in flies are involved with the same metabolic pathways that underlie response to excess sugar in humans. The contrasting genotypes of *Drosophila* species adapted to different natural diets can be harnessed to enhance the value of flies to studies of human metabolism and disease.

## Supporting information

S1 Table Composition of the artificial diets. (DOCX)

S2 Table Sequencing statistics for PE-reads samples of the three species raised on HPLS, EPS, and LPHS. (DOCX)

S3 Table Spearman’s correlation coefficient between replicates. Coefficients were calculated using R with raw counts from cuffdiff outputs.
(DOCX)

S4 Table List of Gene IDs of *D*. *arizonae*, *D*. *mojavensis* and orthologs for pathway enrichment analysis. Excel file that contains the list of flybase gene IDs, gene symbol, KEGG IDs of Differentially Expressed Genes of *D*. *arizonae* and *D*. *mojavensis*, and the same data for the orthologs of *D*. *melanogaster* to execute the pathway enrichment analysis.
(XLSX)

S5 Table Sequences of primers used for RT-qPCRs. Excel file that contains the sequences of each set of primers used to make the RT-qPCRs for validation of RNA-Seq data.
(XLSX)

S6 Table Gene function of differentially expressed genes of *D*. *arizonae* through diets. Protein domains and associated function were obtained from orthologs of *D*. *mojavensis* in FlyBase (http://flybase.org). Red genes also changed in *D*. *mojavensis* diets.
(DOCX)

S7 Table Gene function of differentially expressed genes of *D*. *mojavensis* through diets. Protein domains and associated function were obtained from FlyBase (http://flybase.org). Red genes also changed in *D*. *arizonae* diets.
(DOCX)

S8 Table Analysis of KEGG pathways. Excel file that contains the data generated by “Annotate” and “Identify” programs of KOBAS 2.0 web tool. Analysis was performed using the default options as hypergeometric test/Fisher's exact test for statistical method and a Benjamini and Hochberg FDR-correction method. The significantly represented pathways are marked in red and can be viewed with the associated hyperlinks.
(XLSX)
