# Supplementary material for: Transcriptional responses of ecologically diverse Drosophila species to larval diets differing in relative sugar and protein ratios
Source: PLoS One. 2017 Aug 23;12(8):e0183007. doi: 10.1371/journal.pone.0183007 (PMC5568408; doi:10.1371/journal.pone.0183007)
Supplement: S1 Table — (DOCX) [file pone.0183007.s001.docx]

**S1 Table. Composition of artificial diets.**

|  | **HPLS** | **EPS** | **LPHS** |
| --- | --- | --- | --- |
| **Sucrose (g)** | 8 | 20 | 32 |
| **Active dry yeast (g)** | 32 | 20 | 8 |
| **Yellow cornmeal (g)** | 9 | 9 | 9 |
| **Distilled water (ml)** | 200 | 200 | 200 |
| **Agar (g)** | 1 | 1 | 1 |
| **Ethanol 100% (ml)** | 4.5 | 4.5 | 4.5 |
| **Methyl paraben (g)** | 0.45 | 0.45 | 0.45 |
| **Index protein:sugar** | 0.43 | 0.20 | 0.10 |
