# Supplementary material for: Transcriptional responses of ecologically diverse Drosophila species to larval diets differing in relative sugar and protein ratios
Source: PLoS One. 2017 Aug 23;12(8):e0183007. doi: 10.1371/journal.pone.0183007 (PMC5568408; doi:10.1371/journal.pone.0183007)
Supplement: S2 Table — (DOCX) [file pone.0183007.s002.docx]

**S2 Table. Sequencing statistics for PE-reads samples of the three species raised on HPLS, EPS, and LPHS.**

| **Samples** | **Input*** | **Mapped left reads**** | **Mapped right reads**** |
| --- | --- | --- | --- |
| Dmel_HPLS1 | 14607012 | 13191475 | 13222725 |
| Dmel_HPLS2 | 42116527 | 38425890 | 37863081 |
| Dmel_EPS1 | 22936609 | 20864533 | 20852066 |
| Dmel_EPS2 | 20210922 | 18490146 | 18250477 |
| Dmel_LPHS1 | 15152336 | 13667469 | 13657229 |
| Dmel_LPHS2 | 18956811 | 17437037 | 17241849 |
| Darz_HPLS1 | 17643204 | 13049332 | 13076385 |
| Darz_HPLS2 | 14435525 | 10945111 | 10800078 |
| Darz_EPS1 | 14288405 | 10786834 | 10812945 |
| Darz_EPS2 | 11816693 | 9144242 | 9029733 |
| Darz_LPHS1 | 14724324 | 11117252 | 11116931 |
| Darz_LPHS2 | 15271810 | 11788018 | 11646258 |
| Dmoj_HPLS1 | 15998615 | 14078223 | 14087555 |
| Dmoj_HPLS2 | 15203368 | 13688308 | 13520944 |
| Dmoj_EPS1 | 17305352 | 15246243 | 15274406 |
| Dmoj_EPS2 | 12406720 | 11142796 | 11008257 |
| Dmoj_LPHS1 | 15497412 | 13674082 | 13636723 |
| Dmoj_LPHS2 | 18956811 | 17437037 | 17241849 |

* Raw data obtained with HiSeq^TM^ 2000 Sequencing System from Illumina in a 2 X 100 paired-end reads format.

****** Mapped reads using bowtie2/tophat with standard options
