# Supplementary material for: Transcriptional responses of ecologically diverse Drosophila species to larval diets differing in relative sugar and protein ratios
Source: PLoS One. 2017 Aug 23;12(8):e0183007. doi: 10.1371/journal.pone.0183007 (PMC5568408; doi:10.1371/journal.pone.0183007)
Supplement: S3 Table — Coefficients were calculated using R with raw counts from cuffdiff outputs. (DOCX) [file pone.0183007.s003.docx]

**S3 Table. Spearman’s correlation coefficient between replicates. Coefficients were calculated using R with raw counts from cuffdiff outputs.**

|  | | Spearman’s correlation coefficient |
| --- | --- | --- |
| *D. melanogaster* | | |
| Dmel_HPLS1 | | 0.97 |
| Dmel_HPLS2 |  |  |
| Dmel_EPS1 | | 0.97 |
| Dmel_EPS2 | |  |
| Dmel_LPHS1 | | 0.98 |
| Dmel_LPHS2 | |  |
| *D. arizonae* | | |
| Darz_HPLS1 | | 0.97 |
| Darz_HPLS2 | |  |
| Darz_EPS1 | | 0.97 |
| Darz_EPS2 | |  |
| Darz_LPHS1 | | 0.97 |
| Darz_LPHS2 | |  |
| *D. mojavensis* | | |
| Dmoj_H1_count | | 0.98 |
| Dmoj_H2_count | |  |
| Dmoj_E1_count | | 0.98 |
| Dmoj_E2_count | |  |
| Dmoj_L1_count | | 0.98 |
| Dmoj_L2_count | |  |
