# Supplementary material for: Transcriptional responses of ecologically diverse Drosophila species to larval diets differing in relative sugar and protein ratios
Source: PLoS One. 2017 Aug 23;12(8):e0183007. doi: 10.1371/journal.pone.0183007 (PMC5568408; doi:10.1371/journal.pone.0183007)
Supplement: S6 Table — Protein domains and associated function were obtained from orthologs of D. mojavensis in FlyBase (http://flybase.org). Red genes also changed in D. mojavensis diets. (DOCX) [file pone.0183007.s006.docx]

**S6 Table. Gene function of differentially expressed genes of *D. arizonae* through diets. Protein domains and associated function were obtained from orthologs of *D. mojavensis* in FlyBase (http://flybase.org). Red genes also changed in *D. mojavensis* diets.**

| Tested id | *D. mojavensis* Ortholog (Symbol) | Enzyme/Protein Domains | | Associated Function |
| --- | --- | --- | --- | --- |
| *Genes that were up-regulated in higher sugar versus protein diets.* | | | | |
| XLOC_000511 | Dmoj\GI21508 | Ethanolamine-phosphate phospholyase*/Aminotransferase class-III. | Transaminase activity. | |
| XLOC_001474 | Dmoj\GI17345 | ND | ND | |
| XLOC_002105 | Dmoj\GI23729 | Trehalose 6-phosphate phosphatase* | Catalytic activity (trehalose biosynthetic process). | |
| XLOC_002235 | ND |  |  | |
| XLOC_002396 | ND |  |  | |
| XLOC_002713 | Dmoj\GI19195 | ND | ND | |
| XLOC_002857 | Dmoj\GI19033 | ND | ND | |
| XLOC_003916 | Dmoj\GI13179 | Alkaline phosphatase* | Phosphatase activity (metabolic process). | |
| XLOC_003964 | Dmoj\GI12135 | Alkaline phosphatase* | Phosphatase activity (metabolic process). | |
| XLOC_004041 | Dmoj\GI12003 | Solute carrier family 5-member 8/12 (sodium-coupled monocarboxylate transporter) * | Transporter activity (transmembrane transport). | |
| XLOC_005297 | Dmoj\GI23529 | ND | ND | |
| XLOC_005853 | Dmoj\GI24787 | ND | ND | |
| XLOC_007016 | Dmoj\GI12627 | Insect cuticle protein | Structural constituent of cuticle. | |
| XLOC_007196 | Dmoj\G6pd (Dmoj\GI11107) | Glucose-6-phosphate dehydrogenase* | Glucose-6-phosphate dehydrogenase activity (oxidation-reduction process and glucose metabolic process). | |
| XLOC_009333 | ND |  |  | |
| XLOC_009405 | Dmoj\GI20074 | ND | ND | |
| XLOC_009920 | Dmoj\GI10465 | ND | ND | |
| XLOC_010823 | Dmoj\GI17164 | ND | ND | |
| XLOC_011069 | Dmoj\GI15007 | Eukaryotic translation initiation factor 4E binding protein 2* | Eukaryotic initiation factor 4E binding (negative regulation of translational initiation). | |
| XLOC_011170 | Dmoj\GI20827 | ND | ND | |
| XLOC_011173 | Dmoj\GI20802 | Carbohydrate binding module family 20; Carbohydrate-binding-like fold; Glycerophosphoryl diester phosphodiesterase; Immunoglobulin-like fold; PLC-like phosphodiesterase, TIM beta/alpha-barrel domain. | Glycerophosphodiester phosphodiesterase activity; starch binding (lipid metabolic process and glycerol metabolic process). | |
| XLOC_011578 | Dmoj\GI20954 | Glucuronosyltransferase* | Transferase activity, transferring hexosyl groups (metabolic process). | |
| XLOC_012755 | Dmoj\GI15376 | Choline dehydrogenase*** | Choline dehydrogenase activity; flavin adenine dinucleotide binding (oxidation-reduction process and alcohol metabolic process). | |
| XLOC_013006 | Dmoj\GI13641 | DNA/RNA non-specific endonuclease; Extracellular Endonuclease, subunit A. | Metal ion binding; hydrolase activity; nucleic acid binding. | |
| XLOC_013101 | Dmoj\GI18430 | Solute carrier family 6 member (neurotransmitter transporter, amino acid)* | Neurotransmitter:sodium symporter activity (neurotransmitter transport). | |
| XLOC_013116 | Dmoj\GI15788 | ND | ND | |
| *Genes that were down-regulated in higher sugar versus protein diets.* | | | | |
| XLOC_000655 | Dmoj\GI15562 | Glucose-ribitol dehydrogenase;  NAD(P)-binding domain; Short-chain dehydrogenase/reductase SDR. | Oxidoreductase activity (metabolic process). | |
| XLOC_000804 | Dmoj\GI22106 | Single domain Von Willebrand factor type C. | ND | |
| XLOC_001016 | Dmoj\GI18125 | Acyl-CoA N-acyltransferase; FR47-like; GNAT domain. | N-acetyltransferase activity. | |
| XLOC_001917 | Dmoj\Adh2 (Dmoj\GI17643) | Alcohol dehydrogenase* | Alcohol dehydrogenase (NAD) activity (oxidation-reduction process and alcohol metabolic process). | |
| XLOC_002100 | Dmoj\GI23785 | Sarcosine dehydrogenase* | Oxidoreductase activity; aminomethyltransferase activity (oxidation-reduction process and glycine catabolic process). | |
| XLOC_002812 | Dmoj\GI19059 | Insect cuticle protein. | Structural constituent of cuticle. | |
| XLOC_002934 | Dmoj\GI18904 | AMP-binding enzyme C-terminal domain; AMP-dependent synthetase/ligase. | Catalytic activity (metabolic process). | |
| XLOC_003411 | Dmoj\GI12534 | Purine-nucleoside phosphorylase* | Purine-nucleoside phosphorylase activity (nucleoside metabolic process). | |
| XLOC_004163 | ND |  |  | |
| XLOC_004667 | Dmoj\GI10386 | EF-Hand 1, calcium-binding site, domain. | Calcium ion binding. | |
| XLOC_004906 | Dmoj\GI24339 | Actin beta/gamma 1* | Structural constituent of cytoskeleton.** | |
| XLOC_005117 | Dmoj\GI23906 | CHK kinase-like; Protein kinase-like domain; Protein of unknown function DUF227. | Transferase activity, transferring phosphorus-containing groups. | |
| XLOC_005120 | Dmoj\GI23912 | CHK kinase-like; Protein kinase-like domain; Protein of unknown function DUF227. | Transferase activity, transferring phosphorus-containing groups. | |
| XLOC_005171 | Dmoj\GI23687 | CHK kinase-like; Protein kinase-like domain; Protein of unknown function DUF227. | Transferase activity, transferring phosphorus-containing groups. | |
| XLOC_005172 | Dmoj\GI23685 | CHK kinase-like; Protein kinase-like domain; Protein of unknown function DUF227. | Transferase activity, transferring phosphorus-containing groups. | |
| XLOC_005173 | Dmoj\GI23684 | CHK kinase-like; Protein kinase-like domain; Protein of unknown function DUF227. | Transferase activity, transferring phosphorus-containing groups. | |
| XLOC_005256 | Dmoj\GI24064 | Aldo/keto reductase subgroup; NADP-dependent oxidoreductase domain. | Oxidoreductase activity (oxidation-reduction process). | |
| XLOC_005332 | Dmoj\GI24124 | Carboxylesterase 1* | Cholinesterase activity. | |
| XLOC_005334 | Dmoj\GI23478 | Alpha/Beta hydrolase fold; Carboxylesterase type B, active site; Carboxylesterase, type B. | Hydrolase activity.** | |
| XLOC_005762 | Dmoj\GI22970 | General substrate transporter; Major facilitator superfamily domain. | Transmembrane transporter activity (transmembrane transport). | |
| XLOC_005823 | Dmoj\GI24737 | Domain of unknown function DUF243. | ND | |
| XLOC_005828 | Dmoj\GI24780 | Kazal domain | ND | |
| XLOC_006506 | Dmoj\GI12994 | Insect cuticle protein | Structural constituent of cuticle. | |
| XLOC_006546 | Dmoj\GI12992 | Insect cuticle protein | Structural constituent of cuticle. | |
| XLOC_006558 | ND |  |  | |
| XLOC_007799 | Dmoj\GI24412 | Phosphatidylethanolamine-binding protein PEBP. | ND | |
| XLOC_008427 | Dmoj\GI19553 | Defensin, invertebrate/fungal; Knottin, scorpion toxin-like. | ND | |
| XLOC_008657 | Dmoj\GI23443 | Aminomethyltransferase* | Aminomethyltransferase activity (glycine catabolic process). | |
| XLOC_008841 | Dmoj\GI11539 | Hemocyanin/hexamerin, Immunoglobulin E-set. | ND | |
| XLOC_009153 | Dmoj\GI19361 | Attacin, C-terminal | ND | |
| XLOC_009261 | Dmoj\GI15361 | ND | ND | |
| XLOC_011121 | Dmoj\GI17521 | ND | ND | |
| XLOC_011609 | Dmoj\GI21292 | AMP-binding enzyme C-terminal domain; AMP-binding, conserved site; AMP-dependent synthetase/ligase. | Catalytic activity (metabolic process). | |
| XLOC_011656 | Dmoj\GI15819 | Epidermal growth factor-like domain. | Receptor activity.** | |
| XLOC_012375 | Dmoj\GI15744 | CAP domain; Cysteine-rich secretory protein, allergen V5/Tpx-1-related. | ND | |
| XLOC_012790 | Dmoj\GI24228 | Selenium-binding protein 1* | Selenium binding. | |
| XLOC_012803 | Dmoj\Xdh (Dmoj\GI23360) | Xanthine dehydrogenase/oxidase* | FAD-binding; 2 iron, 2 sulfur cluster binding; UDP-N-acetylmuramate dehydrogenase activity; molybdopterin cofactor binding; electron carrier activity; xanthine dehydrogenase/oxidase activity (oxidation-reduction process). | |
| XLOC_012811 | Dmoj\GI23350 | Cytochrome P450, E-class, group I. | Oxidoreductase activity, acting on paired donors, with incorporation or reduction of molecular oxygen; iron/heme binding (oxidation-reduction process). | |

*****Obtained from Kyoto Encyclopedia of Genes and Genomes (<http://www.genome.jp/kegg/>).

**Obtained from PANTHER Classification System using *D. melanogaster*’s ortholog ID (<http://pantherdb.org>).
