# Supplementary material for: Transcriptional responses of ecologically diverse Drosophila species to larval diets differing in relative sugar and protein ratios
Source: PLoS One. 2017 Aug 23;12(8):e0183007. doi: 10.1371/journal.pone.0183007 (PMC5568408; doi:10.1371/journal.pone.0183007)
Supplement: S7 Table — Protein domains and associated function were obtained from FlyBase (http://flybase.org). Red genes also changed in D. arizonae diets. (DOCX) [file pone.0183007.s007.docx]

**S7 Table. Gene function of differentially expressed genes of *D. mojavensis* through diets.** Protein domains and associated function were obtained from FlyBase (http://flybase.org). Red genes also changed in *D. arizonae* diets.

| Gene_ID | Gene_symbol | Enzyme/Protein Domains | Associated Function |
| --- | --- | --- | --- |
| *Genes that were up-regulated in higher sugar versus protein diets.* | | | |
| FBgn0068806 | Dmoj\G6pd (Dmoj\GI11107) | Glucose-6-phosphate 1-dehydrogenase (G6PD)* | Glucose-6-phosphate dehydrogenase activity (oxidation-reduction process and glucose metabolic process). |
| FBgn0132887 | Dmoj\GI10121 | Acetylcholinesterase* | Hydrolase activity, acting on ester bonds (metabolic process, synaptic transmission).** |
| FBgn0133229 | Dmoj\GI10465 | ND | ND |
| FBgn0133248 | Dmoj\GI10484 | Stearoyl-CoA desaturase (SCD)* | Oxidoreductase activity, acting on paired donors, with oxidation of a pair of donors resulting in the reduction of molecular oxygen to two molecules of water (oxidation-reduction process and lipid metabolic process). |
| FBgn0133646 | Dmoj\GI10883 | ND | ND |
| FBgn0133735 | Dmoj\GI10972 | HAD-like domain; HAD-superfamily hydrolase, subfamily IIA; Nitrophenylphosphatase-like domain. | Hydrolase activity (metabolic process).** |
| FBgn0133832 | Dmoj\GI11069 | 6-phosphogluconate dehydrogenase (PGD)* | NADP-binding; Phosphogluconate dehydrogenase (decarboxylating) activity (pentose-phosphate shunt). |
| FBgn0134664 | Dmoj\GI11906 | BCL2/adenovirus E1B 19 kDa protein-interacting protein 3 (BNIP3)* | Positive regulation of apoptotic process. |
| FBgn0134723 | Dmoj\GI11966 | Insect allergen-related. | ND |
| FBgn0135110 | Dmoj\GI12353 | Argininosuccinate lyase (argH) * | Argininosuccinate lyase activity (arginine biosynthetic process via ornithine). |
| FBgn0135293 | Dmoj\GI12536 | Protein of unknown function DUF725 | ND |
| FBgn0136396 | Dmoj\GI13641 | DNA/RNA non-specific endonuclease; Extracellular Endonuclease, subunit A. | Metal ion binding; hydrolase activity; nucleic acid binding. |
| FBgn0137370 | Dmoj\GI14619 | ND | ND |
| FBgn0137550 | Dmoj\GI14799 | Amino acid transporter, transmembrane. | Transmembrane transporter activity (ion transport and amino acid transport).** |
| FBgn0137636 | Dmoj\GI14885 | Nuclear phosphoprotein p8, DNA binding. | ND |
| FBgn0137747 | Dmoj\GI14996 | Eukaryotic translation initiation factor 4E binding protein 2* | Eukaryotic initiation factor 4E binding (negative regulation of translational initiation). |
| FBgn0137757 | Dmoj\GI15007 | Eukaryotic translation initiation factor 4E binding protein 2* | Eukaryotic initiation factor 4E binding (negative regulation of translational initiation). |
| FBgn0137836 | Dmoj\GI15086 | Cystine-knot cytokine; PDGF/VEGF domain. | Growth factor activity. |
| FBgn0138069 | Dmoj\GI15320 | Disks large 1; PDZ domain. | Nucleotide kinase activity; receptor binding ([nucleobase-containing compound metabolic process](http://pantherdb.org/panther/category.do?categoryAcc=GO:0006139), cell-cell signaling, cellular component organization, system development).** |
| FBgn0138326 | Dmoj\GI15577 | TNFR/NGFR cysteine-rich region. | Receptor activity (cell communication, ectoderm development, cellular defense response).** |
| FBgn0138336 | Dmoj\GI15587 | ND | ND |
| FBgn0139069 | Dmoj\GI16321 | Single domain Von Willebrand factor type C | ND |
| FBgn0139388 | Dmoj\GI16640 | Glycoside hydrolase, family 22, conserved site; Lysozyme-like domain. | ND |
| FBgn0140002 | Dmoj\GI17258 | Chitinase II; Chitinase insertion domain; Glycoside hydrolase, catalytic domain, family 18, superfamily; Imaginal disc growth factor. | Chitinase activity (carbohydrate metabolic process and chitin catabolic process). |
| FBgn0140088 | Dmoj\GI17344 | ND | ND |
| FBgn0140197 | Dmoj\GI17453 | ND | ND |
| FBgn0140438 | Dmoj\GI17697 | C-type lectin fold, conserved site; C-type lectin-like. | Carbohydrate binding. |
| FBgn0141630 | Dmoj\GI18891 | Chitinase* | Chitin binding; chitinase activity (carbohydrate metabolic process and chitin catabolic process). |
| FBgn0141675 | Dmoj\GI18936 | ND | ND |
| FBgn0142128 | Dmoj\GI19390 | Immune-induced protein Dim. | ND |
| FBgn0142129 | Dmoj\GI19391 | ND | ND |
| FBgn0142179 | Dmoj\GI19441 | Gamma-butyrobetaine dioxygenase* | Oxidoreductase activity (oxidation-reduction process). |
| FBgn0142332 | Dmoj\GI19595 | Actin beta/gamma 1* | Structural constituent of cytoskeleton (cellular process, developmental process, protein transport, vesicle-mediated transport, cellular component organization).** |
| FBgn0142639 | Dmoj\GI19902 | ND | ND |
| FBgn0142712 | Dmoj\GI19975 | Zinc finger C2H2-type/integrase DNA-binding domain. | Metal ion binding; nucleic acid binding. |
| FBgn0142851 | Dmoj\GI20115 | Immune-induced protein Dim. | ND |
| FBgn0143523 | Dmoj\GI20788 | General substrate transporter; Major facilitator superfamily domain; Sugar transporter, conserved site; Sugar/inositol transporter. | Substrate-specific transmembrane transporter activity (transmembrane transport). |
| FBgn0143571 | Dmoj\GI20837 | Attacin, N/C-terminal. | ND |
| FBgn0143688 | Dmoj\GI20954 | Glucuronosyltransferase (UGT)* | Transferase activity, transferring hexosyl groups (metabolic process). |
| FBgn0143980 | Dmoj\GI21250 | Trypsin* | Serine-type endopeptidase activity (proteolysis). |
| FBgn0144238 | Dmoj\GI21508 | Ethanolamine-phosphate phospho-lyase* | Pyridoxal phosphate binding; transaminase activity. |
| FBgn0144404 | Dmoj\GI21674 | General substrate transporter; Major facilitator superfamily domain; Sugar transporter, conserved site; Sugar/inositol transporter. | Substrate-specific transmembrane transporter activity (transmembrane transport). |
| FBgn0145324 | Dmoj\GI22596 | Metallopeptidase/Peptidase M12A, astacin. | Metalloendopeptidase activity (proteolysis), zinc ion binding. |
| FBgn0145335 | Dmoj\GI22607 | Metallopeptidase/Peptidase M12A, astacin. | Metalloendopeptidase activity (proteolysis), zinc ion binding. |
| FBgn0145346 | Dmoj\GI22618 | Metallopeptidase/Peptidase M12A, astacin. | Metalloendopeptidase activity (proteolysis), zinc ion binding. |
| FBgn0145663 | Dmoj\GI22936 | L-iditol 2-dehydrogenase (SORD)* | Oxidoreductase activity; zinc ion binding (oxidation-reduction process). |
| FBgn0146454 | Dmoj\GI23729 | Trehalose 6-phosphate phosphatase (otsB)* | Catalytic activity (trehalose biosynthetic process). |
| FBgn0146886 | Dmoj\GI24163 | Heat shock protein 70kD 1/8* | Protein folding, response to stress and protein complex biogenesis.** |
| FBgn0147046 | Dmoj\GI24323 | Stearoyl-CoA desaturase (SCD)* | Oxidoreductase activity, acting on paired donors, with oxidation of a pair of donors resulting in the reduction of molecular oxygen to two molecules of water (oxidation-reduction process and lipid metabolic process). |
| FBgn0147161 | Dmoj\GI24439 | Peptidase S1A, chymotrypsin-type (trypsin-like cysteine/serine peptidase domain). | Serine-type endopeptidase activity (proteolysis). |
| FBgn0147292 | Dmoj\GI24570 | Chitin binding domain. | Chitin binding (chitin metabolic process). |
| FBgn0147586 | Dmoj\GI24866 | Solute carrier-family 26/member 11 (sodium-independent sulfate anion transporter)* | Sulfate transmembrane transporter activity (sulfate transport). |
| *Genes that were down-regulated in higher sugar versus protein diets.* | | | |
| FBgn0012567 | Dmoj\Adh2 (Dmoj\GI17643) | Alcohol dehydrogenase* | Alcohol dehydrogenase (NAD) activity (oxidation-reduction process and alcohol metabolic process). |
| FBgn0064059 | Dmoj\Xdh (Dmoj\GI23360) | Xanthine dehydrogenase/ Aldehyde oxidase* | Xanthine dehydrogenase/oxidase activity (oxidation-reduction process). |
| FBgn0132918 | Dmoj\GI10152 | Xanthine dehydrogenase/oxidase* | FAD-binding; 2 iron, 2 sulfur cluster binding; iron ion binding; UDP-N-acetylmuramate dehydrogenase activity; electron carrier activity (oxidation-reduction process). |
| FBgn0133429 | Dmoj\GI10665 | ND | ND |
| FBgn0134299 | Dmoj\GI11539 | Hemocyanin/hexamerin, Immunoglobulin E-set. | [Oxidoreductase activity](http://pantherdb.org/panther/category.do?categoryAcc=GO:0016491) ([lipid metabolic process](http://pantherdb.org/panther/category.do?categoryAcc=GO:0006629)).** |
| FBgn0134449 | Dmoj\GI11690 | ND | ND |
| FBgn0135162 | Dmoj\GI12405 | Chitin binding domain. | Chitin binding (chitin metabolic process). |
| FBgn0135291 | Dmoj\GI12534 | Purine nucleoside phosphorylase* | Purine-nucleoside phosphorylase activity (nucleoside metabolic process). |
| FBgn0135424 | Dmoj\GI12667 | ND | ND |
| FBgn0135451 | Dmoj\GI12694 | Insect cuticle protein | Structural constituent of cuticle. |
| FBgn0135737 | Dmoj\GI12980 | Alkaline phosphatase* | Phosphatase activity (metabolic process). |
| FBgn0135751 | Dmoj\GI12994 | Insect cuticle protein | Structural constituent of cuticle. |
| FBgn0135759 | Dmoj\GI13002 | Cytochrome P450, E-class, group I. | Oxidoreductase activity (paired donors) with incorporation or reduction of molecular oxygen; iron/heme binding (oxidation-reduction process). |
| FBgn0135940 | Dmoj\GI13183 | Polypeptide N-acetylgalactosaminyltransferase* | Transferase activity (carbohydrate metabolic process and protein glycosylation).** |
| FBgn0136286 | Dmoj\GI13531 | ND | ND |
| FBgn0136517 | Dmoj\GI13762 | Hemocyanin, C/N-terminal; Hemocyanin/hexamerin; Immunoglobulin E-set. | Oxidoreductase activity (lipid metabolic process).** |
| FBgn0137352 | Dmoj\GI14601 | Fatty acid hydroxylase | Iron ion binding; oxidoreductase activity (oxidation-reduction process and fatty acid biosynthetic process). |
| FBgn0137379 | Dmoj\GI14628 | Acyltransferase 3; Nose resistant-to-fluoxetine protein, N-terminal. | Transferase activity, transferring acyl groups other than amino-acyl groups. |
| FBgn0137536 | Dmoj\GI14785 | ND | ND |
| FBgn0138311 | Dmoj\GI15562 | Glucose/ribitol dehydrogenase; NAD(P)-binding domain; Short-chain dehydrogenase/reductase SDR. | Oxidoreductase activity (metabolic process). |
| FBgn0138471 | Dmoj\GI15722 | Protein of unknown function DUF725 | ND |
| FBgn0138918 | Dmoj\GI16169 | CHK kinase-like; Protein kinase-like domain; Protein of unknown function DUF227. | Transferase activity, transferring phosphorus-containing groups. |
| FBgn0138920 | Dmoj\GI16171 | Biotinidase, eukaryotic; Carbon-nitrogen hydrolase; Phospholipase C, phosphatidylinositol-specific, Y domain | Hydrolase activity, acting on carbon-nitrogen (but not peptide) bonds, in linear amides; phosphatidylinositol phospholipase C activity (intracellular signal transduction, nitrogen compound metabolic process and lipid metabolic process). |
| FBgn0138921 | Dmoj\GI16172 | Biotinidase, eukaryotic; Carbon-nitrogen hydrolase; Phospholipase C, phosphatidylinositol-specific, Y domain. | Hydrolase activity, acting on carbon-nitrogen (but not peptide) bonds, in linear amides; phosphatidylinositol phospholipase C activity (intracellular signal transduction, nitrogen compound metabolic process and lipid metabolic process). |
| FBgn0139296 | Dmoj\GI16548 | GYR motif. | ND |
| FBgn0139489 | Dmoj\GI16741 | General substrate transporter; Major facilitator superfamily domain; Sugar transporter, conserved site. | Transmembrane transporter activity (transmembrane transport). |
| FBgn0139805 | Dmoj\GI17060 | O-acyltransferase, WSD1, N-terminal. | Diacylglycerol O-acyltransferase activity (glycerolipid biosynthetic process). |
| FBgn0140142 | Dmoj\GI17398 | ND | ND |
| FBgn0140191 | Dmoj\GI17447 | Alkaline-phosphatase-like, core domain; Protein of unknown function DUF229. | Catalytic activity (metabolic process). |
| FBgn0140247 | Dmoj\GI17503 | Immunoglobulin I-set/subtype 2; Metallopeptidase/Peptidase M13, C/N-terminal domain. | Metalloendopeptidase activity (proteolysis). |
| FBgn0140261 | Dmoj\GI17517 | Acyl-CoA N-acyltransferase | ND |
| FBgn0140267 | Dmoj\GI17523 | Glucuronosyltransferase (UGT)* | Transferase activity, transferring hexosyl groups (metabolic process). |
| FBgn0140316 | Dmoj\GI17572 | ND | ND |
| FBgn0140465 | Dmoj\GI17724 | 3,2-trans-enoyl-CoA isomerase, mitocondrial (DCI)* | Catalytic activity (metabolic process). |
| FBgn0140503 | Dmoj\GI17762 | Proteinase inhibitor I2, Kunitz metazoa, conserved site. | Serine-type endopeptidase inhibitor activity. |
| FBgn0140588 | Dmoj\GI17847 | Lipid transport protein, N-terminal, beta-sheet shell; Vitellinogen, beta-sheet N-terminal, superhelical. | Lipid transporter activity (lipid transport). |
| FBgn0140866 | Dmoj\GI18126 | Acyl-CoA N-acyltransferase; FR47-like; GNAT domain. | N-acetyltransferase activity. |
| FBgn0141413 | Dmoj\GI18674 | Cytochrome P450, E-class, group I. | Oxidoreductase activity (paired donors) with incorporation or reduction of molecular oxygen; iron/heme binding (oxidation-reduction process). |
| FBgn0141436 | Dmoj\Mal-A5 (Dmoj\GI18697) | Glycoside hydrolase, catalytic domain, family 13. | Cation binding; catalytic activity (carbohydrate metabolic process). |
| FBgn0141643 | Dmoj\GI18904 | AMP-binding enzyme C-terminal domain; AMP-dependent synthetase/ligase. | Catalytic activity (metabolic process). |
| FBgn0141798 | Dmoj\GI19059 | Insect cuticle protein | Structural constituent of cuticle. |
| FBgn0141814 | Dmoj\GI19075 | Acyl-CoA oxidase* | Acyl-CoA dehydrogenase/ oxidase activity; FAD-binding (fatty acid beta-oxidation). |
| FBgn0142021 | Dmoj\GI19282 | ND | ND |
| FBgn0142026 | Dmoj\GI19287 | Hemocyanin, C/N-terminal; Hemocyanin/hexamerin middle domain; Immunoglobulin E-set; Tyrosinase copper-binding domain; Uncharacterised domain, di-copper centre. | Oxidoreductase activity (metabolic process). |
| FBgn0142249 | Dmoj\GI19511 | Insect cuticle protein | Structural constituent of cuticle. |
| FBgn0142279 | Dmoj\GI19542 | Insect cuticle protein | Structural constituent of cuticle. |
| FBgn0142353 | Dmoj\GI19616 | Kazal domain; Major facilitator superfamily domain, general substrate transporter; Organic anion transporter polypeptide OATP. | Transporter activity (transport). |
| FBgn0142374 | Dmoj\GI19637 | ND | ND |
| FBgn0142514 | Dmoj\GI19777 | ND | ND |
| FBgn0142679 | Dmoj\GI19942 | Hexokinase (HK)* | ATP binding; phosphotransferase activity, alcohol group as acceptor (carbohydrate metabolic process). |
| FBgn0142795 | Dmoj\GI20059 | Tetraspanin, EC2 domain; Tetraspanin/Peripherin. | Receptor activity (cellular process).** |
| FBgn0142964 | Dmoj\GI20228 | Cytochrome P450, E-class, group I. | Oxidoreductase activity (paired donors) with incorporation or reduction of molecular oxygen; iron/heme binding (oxidation-reduction process). |
| FBgn0142965 | Dmoj\GI20229 | Cytochrome P450, E-class, group I. | Oxidoreductase activity (paired donors) with incorporation or reduction of molecular oxygen; iron/heme binding (oxidation-reduction process). |
| FBgn0143363 | Dmoj\GI20628 | ND | ND |
| FBgn0143512 | Dmoj\GI20777 | Protein transport protein SecG/Sec61-beta/Sbh1* | ND |
| FBgn0143637 | Dmoj\GI20903 | Insect cuticle protein | Structural constituent of cuticle. |
| FBgn0143638 | Dmoj\GI20904 | Insect cuticle protein | Structural constituent of cuticle. |
| FBgn0143639 | Dmoj\GI20905 | Insect cuticle protein | Structural constituent of cuticle. |
| FBgn0143677 | Dmoj\GI20943 | Glucuronosyltransferase (UGT)* | Transferase activity, transferring hexosyl groups (metabolic process). |
| FBgn0144022 | Dmoj\GI21292 | AMP-binding enzyme C-terminal domain/conserved site; AMP-dependent synthetase/ligase. | Catalytic activity (metabolic process). |
| FBgn0145204 | Dmoj\GI22476 | Alpha/Beta hydrolase fold; Carboxylesterase type B, active site. | Hydrolase activity, acting on ester bonds; protein binding (metabolic process, cell-cell signaling, cell adhesión, neurological system process, nervous system development, cellular component organization).** |
| FBgn0145301 | Dmoj\GI22573 | CHK kinase-like; Protein kinase-like domain; Protein of unknown function DUF227. | Transferase activity, transferring phosphorus-containing groups. |
| FBgn0145356 | Dmoj\GI22628 | Glucuronosyltransferase (UGT)* | Transferase activity, transferring hexosyl groups (metabolic process). |
| FBgn0145698 | Dmoj\GI22971 | Major facilitator superfamily domain, general substrate transporter. | Transmembrane transporter activity (transmembrane transport). |
| FBgn0145729 | Dmoj\GI23002 | ND | ND |
| FBgn0145801 | Dmoj\GI23074 | Hemocyanin, C/N-terminal; Hemocyanin/hexamerin middle domain; Immunoglobulin E-set; Uncharacterised domain, di-copper centre. | Oxidoreductase activity (lipid metabolic process and transport).** |
| FBgn0145835 | Dmoj\GI23108 | Major facilitator superfamily domain, general substrate transporter. | Transmembrane transport. |
| FBgn0145987 | Dmoj\GI23260 | CHK kinase-like; Protein kinase-like domain; Protein of unknown function DUF227. | Transferase activity, transferring phosphorus-containing groups. |
| FBgn0146077 | Dmoj\GI23350 | Cytochrome P450, E-class, group I. | Oxidoreductase activity, acting on paired donors, with incorporation or reduction of molecular oxygen; iron/heme binding (oxidation-reduction process). |
| FBgn0146169 | Dmoj\GI23443 | Aminomethyltransferase (gcvT)* | Aminomethyltransferase activity (glycine catabolic process). |
| FBgn0146410 | Dmoj\GI23684 | CHK kinase-like; Protein kinase-like domain; Protein of unknown function DUF227. | Transferase activity, transferring phosphorus-containing groups. |
| FBgn0146411 | Dmoj\GI23685 | CHK kinase-like; Protein kinase-like domain; Protein of unknown function DUF227. | Transferase activity, transferring phosphorus-containing groups. |
| FBgn0146434 | Dmoj\GI23709 | Deoxyribonuclease II* | Deoxyribonuclease II activity (DNA metabolic process). |
| FBgn0146510 | Dmoj\GI23785 | Sarcosine dehydrogenase* | Oxidoreductase activity (oxidation-reduction process and glycine catabolic process). |
| FBgn0146631 | Dmoj\GI23906 | CHK kinase-like; Protein kinase-like domain; Protein of unknown function DUF227. | Transferase activity, transferring phosphorus-containing groups. |
| FBgn0146637 | Dmoj\GI23912 | CHK kinase-like; Protein kinase-like domain; Protein of unknown function DUF227. | Transferase activity, transferring phosphorus-containing groups. |
| FBgn0146687 | Dmoj\GI23963 | Calreticulin*; Concanavalin A-like lectin/glucanases superfamily. | Calcium ion binding; unfolded protein binding (protein folding). |
| FBgn0146697 | Dmoj\GI23973 | D-amino-acid oxidase* | D-amino-acid oxidase activity (oxidation-reduction process). |
| FBgn0146847 | Dmoj\GI24124 | Alpha/Beta hydrolase fold; Carboxylesterase, type B; Cholinesterase. | Cholinesterase activity. |
| FBgn0146916 | Dmoj\Obp99c (Dmoj\GI24193) | Pheromone/general odorant binding protein (PBP/GOBP domain). | Odorant binding. |
| FBgn0147062 | Dmoj\GI24339 | Actin beta/gamma 1* | Structural molecule activity (cellular process, cellular component morphogenesis, protein transport, vesicle-mediated transport and cellular component organization).** |
| FBgn0147168 | Dmoj\GI24446 | MD-2-related lipid-recognition domain; Immunoglobulin E-set. | Lipid metabolic process and lipid transport.** |
| FBgn0147319 | Dmoj\GI24597 | ND | ND |
| FBgn0147459 | Dmoj\GI24737 | Domain of unknown function DUF243. | ND |
| FBgn0147462 | Dmoj\GI24740 | Cytochrome P450-family 6* | Oxidoreductase activity, acting on paired donors, with incorporation or reduction of molecular oxygen; iron/heme binding (oxidation-reduction process). |
| FBgn0147502 | Dmoj\GI24780 | Kazal domain. | ND |
| FBgn0147506 | Dmoj\GI24784 | Kazal domain. | ND |
| FBgn0147523 | Dmoj\GI24801 | C-type lectin. | Carbohydrate binding. |
| FBgn0147589 | Dmoj\GI24869 | Solute carrier-family 31/member 1 (copper transporter)* | Copper ion transmembrane transporter activity (copper ion transmembrane transport). |

* Obtained from Kyoto Encyclopedia of Genes and Genomes (<http://www.genome.jp/kegg/>).

** Obtained from PANTHER Classification System using *D. melanogaster*’s ortholog ID (http://pantherdb.org).
